# Tribo-Mechanical Properties of Nanomultilayer TiCN/ZrCN Coatings with Different Carbon Content

**DOI:** 10.3390/ma19071316

**Published:** 2026-03-26

**Authors:** Tetiana Cholakova, Lilyana Kolaklieva, Stefan Kolchev, Kiril Kirilov, Daniela Kovacheva, Evgenia Valcheva, Ekaterina Zlatareva, Christo Bahchedjiev, Roumen Kakanakov, Vasiliy Chitanov

**Affiliations:** 1 Central Laboratory of Applied Physics, Bulgarian Academy of Sciences, 61 St. Petersburg Blvd., 4000 Plovdiv, Bulgaria; ohmic@clapbas.bg (L.K.); st_kolchev@abv.bg (S.K.); ekpepe31@abv.bg (E.Z.); hristobah1@gmail.com (C.B.); ipfban@clapbas.bg (R.K.); vchitanov@gmail.com (V.C.); 2 National Centre of Excellence Mechatronics and Clean Technologies, 8 Blvd. Kliment Ohridski, 1700 Sofia, Bulgaria; 3 Faculty of Physics, Sofia University, 5, Blvd. J. Bourchier, 1164 Sofia, Bulgaria; kirilowk@phys.uni-sofia.bg (K.K.); epv@phys.uni-sofia.bg (E.V.); 4 Institute of General and Inorganic Chemistry, Bulgarian Academy of Sciences, Bl. 11 Acad. Georgi Bonchev Str., 1113 Sofia, Bulgaria; didka@svr.igic.bas.bg

**Keywords:** PVD coatings, TiCN, ZrCN, multilayers, morphology, microstructure, hardness, wear resistance

## Abstract

This work focuses on the study of tribo-mechanical and microstructural properties of TiCN/ZrCN multilayer coatings with a modulation period of 12 nm, obtained by a conventional cathodic arc technique. The coatings were deposited at a temperature of 320 °C using nitrogen and methane reactive gases (N_2_/CH_4_) mixture in three different proportions. Surface morphology, composition, hardness, adhesion, friction and wear behavior were studied using atomic force microscopy, scanning electron microscopy with energy dispersive spectroscopy, X-ray diffraction, Raman spectroscopy, nanoindentation, and scratch and wear tests. The analysis of the coating composition revealed a strict dependence of the carbon content on the CH_4_ flow rate. It was found that the coatings with a carbon content of 14.6 at.% and 15.9 at.% consist of crystalline TiZr (C,N) with the presence of amorphous carbon. All the studied TiCN/ZrCN coatings showed improved tribo-mechanical properties compared to TiN/ZrN multilayers obtained under the same deposition conditions. The highest hardness of 40 GPa was obtained for the coating deposited at a N_2_/CH_4_ flow rate of 370/100 sccm. The lowest wear rate of 3.16 × 10^−6^ mm^3^/N·m under dry sliding conditions was observed in the multilayer coatings deposited at the N_2_/CH_4_ flow rates of 330/140 sccm.

## 1. Introduction

Interest in nanostructured multilayer coatings has been growing rapidly over the last ten years. The conducted studies have shown that through the appropriate selection of individual layers and their arrangement in a multilayer configuration, a significant improvement in mechanical and tribological characteristics can be achieved [1,2,3,4,5]. When materials for a multilayer architecture with improved mechanical properties are selected, their crystal structure and compatibility with the substrate should be taken into account, in addition to the hardness and elastic modulus. These requirements often lead to the development of multilayer coatings with a rather complex architecture, containing several sub-layers, providing better compatibility between the coating and substrate. Among the many coatings developed for industrial applications by different methods, the most studied are the nitrides, carbides and carbonitrides of transition metals. In the last five years coatings such as (Zr,Nb)N and TiN(V,Zr) have been developed by PVD methods and the effect of Nb, Zr and V doping on the properties of ZrN and TiN monolayers was investigated [6,7]. For all of the coatings, an improvement in the microstructure, mechanical and tribological properties was achieved; the (Zr,Nb)N coating with a gradient structure demonstrated higher hardness and lower wear resistance compared to TiZrN and TiVN ones. Carbonitrides with a single transition metal, such as TiCN [8,9,10,11,12,13], CrCN [14,15] and ZrCN [16,17,18], are the most widely used in industrial applications, due to their ability to combine the superior properties of nitrides and carbides. Ti- and Zr-based nitrides and carbides have the same face-centered cubic NaCl-type crystal structures. These compounds exist in a wide range of compositions due to vacancies in the carbon or nitrogen sub-lattice and have a relatively high hardness, depending on the preparation technology. The persistent search for new coatings with improved parameters for multifunctional applications has led to the emergence of a new generation of carbonitrides, in which one or more elements are added to the basic Me(C,N) compound. The developed coatings CrVCN [19], TiAlCN [20], ZrTiCN [21], CrAlCN [22], TiNbZrCN [23] possess better mechanical and tribological properties than the conventional monolayer carbonitrides used. At the same time, researchers’ efforts were focused on the development of multilayer nitride coatings, and those in combination with harder carbonitrides, to obtain coatings with improved hardness, adhesion and wear resistance. For the studied multilayer nitrides, it has been experimentally found [24,25,26] that increasing the number of interfaces in the multilayer structure generates more barriers for dislocation movement and crack propagation. In addition, an increase in hardness and Young’s modulus, and hence in resistance to plastic deformation, was observed. It has been reported that the addition of certain amounts of carbon to multilayer nitride coatings [27,28,29,30,31] improves their structural and mechanical properties and affects the wear and friction coefficient of the coatings.

Of great interest are multilayer coatings with a modulation period thickness much less than 100 nm, which brings their properties closer to those of superlattices [32,33,34]. One of the important advantages of these coatings is the presence of a large number of interfaces that intercept dislocation movement and crack propagation through the depth of the coating. However, only a few studies have been carried out on the deposition and characterization of transition metal carbonitrides with a nanomultilayer structure. Advanced CrCN/ZrCN coatings with a bilayer period of 20 nm have been developed [35] and the influence of the carbon content on the microstructure and tribo-mechanical properties was reported. These coatings showed an optimal combination of mechanical and tribological properties at a carbon concentration of 4.2 at.% (hardness of 27.9 GPa, critical load of 41.0 N and wear rate of 1.4 × 10^−6^ mm^3^/Nm). In another study [36], a series of nanometer TiCN/ZrCN coatings with constant carbon content and different bilayer thicknesses were studied. The best combination of tribo-mechanical properties was found in the coating with a bilayer period of 10 nm: hardness 34.8 GPa, adhesion strength of 51 N and friction coefficient of approximately 0.3. In our previous work [37], we reported on TiCN/ZrCN nanomultilayer coatings with a bilayer period of 12 nm and a carbon content of 16 at.% within the periodic structure. The obtained results for the mechanical properties of this coating are superior to those mentioned above, featuring a hardness of 37 GPa and an adhesion strength of 63 N. As an optimal composition of nanoscale multilayer TiCN/ZrCN coatings that combines the best structural, mechanical, and tribological properties has not yet been identified, we are motivated to continue optimizing the modulated structure.

The objective of this work was to develop wear-resistant TiCN/ZrCN coatings with enhanced tribo-mechanical properties by incorporating varying carbon concentrations into a previously created TiN/ZrN nanomultilayer structure using cathodic arc deposition.

## 2. Experimental Materials and Methods

### 2.1. Coatings Preparation

The experiments for deposition of the coatings were performed using a conventional unfiltered cathodic arc evaporation system (CAES), manufactured by Stivak Ltd. (Plovdiv, Bulgaria). The TiCN/ZrCN multilayer coatings were obtained using two cathodes mounted oppositely on the side walls of the cylindrical working chamber. Titanium (Ti) and zirconium (Zr) targets with a purity of 99.99% and a diameter of 70 mm were used. The carbon content in the coatings was varied by changing the reactive gas nitrogen (99.9999) to methane (99.995) ratio in the following proportions of N_2_/CH_4_: 85%/15%, 80%/20% and 70%/30%. All the multilayer coatings were deposited by alternately rotating the specimens between the plasma of Ti and Zr targets. A rotation speed of 10 rpm was used to obtain coatings with the same modulation period. Polished austenitic stainless steel (SS316) EN 1.4401 and high-speed steel (HSS) EN 1.3343 materials in the form of HSS coupons with diameter of 20 mm and thickness of 5 mm and square SS plates with dimensions of 10 mm × 10 mm were used as the substrates for deposition of the coatings. Prior to the deposition, the substrates were ultrasonically cleaned in alkaline degreaser (Borer Chemie AG, Zuchwil, Switzerland), rinsed in deionized water followed by treatment in hot isopropanol, and dried. After that they were fixed on a rotating holder in vacuum chamber at a distance of 200 mm from the targets and the chamber was pumped down to a base pressure of about 3.5 × 10^−3^ Pa. Table 1 presents the technological parameters of the coatings deposition in more detail.

An additional cleaning of the substrates was performed by etching with titanium ions in Ar atmosphere using a DC bias of −1000 V. The working pressure of 0.3 Pa and a temperature of specimens at 320 °C were kept the same during all the coating deposition processes. A negative bias of 80 V was applied to the substrates during formation of all the coatings. A TiZrCN gradient structure was deposited by stepwise decreasing the nitrogen flow and increasing the methane flow to obtain transition layer between the TiZrN and the multilayer modulated TiCN/ZrCN structure with a bilayer period of 12 nm. Single-layer coatings of TiCN and ZrCN were prepared as reference samples under the same process conditions to determine the deposition rate of individual sub-layers in the bilayer period composition.

### 2.2. Characterization Methods

The thickness of the coatings was determined in accordance with the international standard ISO 26423:2009(E) [38] using a compact Calotest CAT^2^ (Anton Paar, Graz, Austria) equipped with light microscopy (Anton Paar Tritec SA, Corcelles, Switzerland) with a magnification of 10×. A depression with the shape of a spherical crater (calotte) was abraded into both the coating and the substrate upon adding diamond suspension to the contact zone “steel ball”–“coated sample”. The coating thickness was calculated by specialized Video Software Version 9.0.12 after microscopic examination of the obtained crater.

Atomic Force Microscopy analysis of surface roughness was performed on an MFP-3D Origin microscope manufactured by Asylum Research, Oxford Instruments (Santa Barbara, CA, USA). The measurements were made in tapping mode, with a scan area of 5 × 5 μm, a line scan rate of 0.5 Hz, and an image resolution of 256 × 256 points. Silicon probes (Opus-160AC-NA, OPUS by µmasch, Innovative Solutions Bulgaria Ltd., Sofia, Bulgaria) with a cantilever length of 160 μm and a reflective Al coating on the backside were used in the experiments. The probes have a nominal cantilever resonance frequency of 300 kHz and a typical force constant of 26 N/m. The nominal tip radius for these probes is <7 nm. Before analysis, the images were aligned along the lines using the median difference method and levelled at three points. Gwyddion 2.65 software was used for the image analysis. Evaluation of topography and surface roughness parameters was done using the obtained images.

A Hitachi SU-5000 scanning electron microscope (Hitachi, Tokyo, Japan) equipped with an energy dispersive spectroscope (Thermo Scientific, Waltham, MA, USA) was used to investigate surface morphology of the coatings, and elemental composition on the surface and in the cross-section. An ion milling machine IM 4000 Plus (Hitachi, Tokyo, Japan) was used to form the cross-section in the coated samples. This procedure was performed using an ion gun in a high-purity argon environment, at an accelerating voltage of 6 kV and a discharge voltage of 1.5 kV, with a duration allowing access to the substrate. Surface and cross-sectional images were obtained in secondary electrons (morphology contrast) and backscattered electrons (density contrast) at an operated voltage of 5 kV and 15 kV, respectively.

The phase composition and structural parameters of coatings were studied with a Bruker D8 Advance X-ray Diffractometer (Bruker AXS, Karlsruhe, Germany) in a Bragg–Brentano configuration using CuKα radiation (λ = 1.5418Å) and LynxEye detector (Bruker AXS, Karlsruhe, Germany). The data were collected in the range 10–80° 2θ with a constant step of 0.03 °2θ and a counting time of 52 sec./step. Phase identification was performed with EVA software (version V4) with the aid of the ICDD-PDF (2024) database. The unit cell parameters and crystallite size were calculated using Topas 4.2, employing the fundamental parameters approach for the diffraction experiment.

The Raman measurements were carried out in backscattering geometry using a micro-Raman HORIBA Jobin Yvon (HORIBA Jobin Yvon, Longjumeau, France) LabRAM HR 800 visible spectrometer equipped with a Peltier-cooled CCD detector Synapse (Horiba JobinYvon, France) with He–Ne (633 nm wavelength and 0.5 mW) laser excitation. The studied frequency shift range was of 100–2000 cm^−1^.

Mechanical properties were investigated using a nanoindentation module, equipped with a diamond Berkovich indenter (CSM Instruments, CH-2034 Peseux, Switzerland). The angle between the centerline and the three faces of the pyramid tip is 65.3°. The nanoindentation module is part of the Compact Platform CPX (MHT/NHT) system (CSM Instruments-Anton Paar GmbH, Graz, Austria), on which a module for conducting adhesion and wear tests is also located. The measurements were performed in loading interval from 20 mN to 500 mN. The Oliver & Pharr method was applied for determination of nanohardness (H), modulus of elasticity (E) and elastic recovery (W_e_) by means of Anton Paar’s software version 6.0.35.

Bruker’s UMT Universal Mechanical Tester (Bruker Nano Surfaces Division, San Jose, CA, USA) with two drive modules simulating linear and rotational motions was used for conducting the adhesion and wear tests. The measurements were performed by positioning the upper sample (indenter or ball) over the bottom sample surface based on a preliminary programmed pattern, speed, and normal force. UMT Viewer was used to display and process the test results.

The adhesion/cohesion strength of the coatings to the substrate material was evaluated using linear module equipped with a spherical Rockwell indenter with a tip radius of 200 μm. The device is equipped with an optical microscope to display the scratch deformation modes and to associate them with the applied load. During the scratch test, the friction force, the penetration depth and the course of the friction coefficient are recorded as secondary data together with the normal force (Fz). After the tests are completed, the traces are analyzed under an optical microscope for specific damages such as cracking, spallation, chipping or delamination of the coatings. Critical loads (Lc) related to the practical bond strength and damage resistances of the coating or coating/substrate system were established for the studied coatings.

Tribological experiments were done in ball-on-disk rotation mode with wear track radius of 2 mm, load of 5 N, sliding speed of 150 rpm and test distance of 500 m (corresponds to 39,809 revolutions). A sapphire ball (Al_2_O_3_) with hardness of 19 GPa and a diameter of 6.35 mm was used as a counterbody. All the tests were performed under the same dry sliding conditions at room temperature (23 ± 2) °C and relative humidity (RH) of 30 ± 10%. The evolution of coefficient of friction (CoF) as a function of sliding time was recorded during the test. The track profiles obtained were used for the calculation of wear rate. Scanning electron microscopy and energy-dispersive x-ray spectroscopy analysis were used to determine the wear mechanisms and identify the elements within the track, respectively.

## 3. Results and Discussion

### 3.1. Coating Thickness and Surface Roughness

Since the bilayer period calculated based on the results of the TiCN and ZrCN monolayers was very small, only the full thickness of the coatings was determined. The calculations were done using specialized video software version 9.0.12 after microscopic examination of the resulting spherical crater. For the correct calculation of the coating thickness the following necessary conditions were met: smooth and flat surface of the substrate and the coating; well-controlled grinding of the crater; round and well-defined perimeter of the crater obtained after the test. The values of thickness obtained by the Calotest method for the coatings deposited under different ratios of N_2_/CH_4_ were in the range of 4.8–6.4 μm.

**Figure 1:**
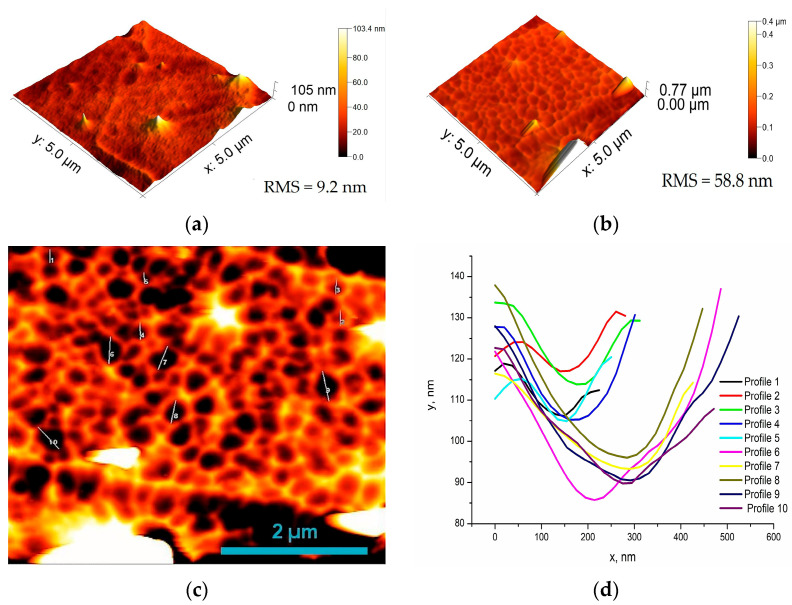
AFM topography images of the coating surfaces: (**a**) TiN/ZrN_ml; (**b**) TiCN/ZrCN_ml (#E21); 2D AFM topography image of TiCN/ZrCN_ml with marked pits (**c**), and their profiles (**d**).

Surface roughness is a significant parameter affecting the accuracy of the experimental results [39,40], especially in the study of mechanical properties by nanoindentation [41]. AFM topography images of the studied TiN/ZrN and TiCN/ZrCN multilayer coatings obtained at the N_2_/CH_4_ flow rate of 330/140 sccm, for which the lowest and highest root mean square roughness values were measured, are presented in Figure 1. The 3D images (Figure 1a,b) show that the surface of both the coatings are dense, without cracks. A small number of sharp convex defects and many pits of various sizes are visible, scattered across the surface. These features of the surfaces are typical for the cathodic arc deposition technology. They are due to the droplet phase originated from the targets, which contributes to increases in the average roughness of the coatings.

According to the measured values, the root mean square (RMS) roughness values of the TiN/ZrN and TiCN/ZrCN multilayer structures over the entire measured area are 9.2 nm and 58.8 nm, respectively. The obtained results are significantly lower than those reported in [35] for CrCN/ZrCN coatings obtained by CAD technology, where an average roughness of (69 ± 19) nm was calculated. Slightly lower values for RMS roughness were measured in the flat regions of the topography images: 6 nm and 20 nm for the nitrides and carbonitrides, respectively. Figure 1c presents a 2D topography image of the TiCN/ZrCN coating with marked pits of various sizes distributed on the surface. The profiles of ten representative pits are presented in Figure 1d. The results of the profile analysis showed that the pits registered on the coating surface have a depth in the range of 10–40 nm and a width of 160–470 nm. The surface morphology of multilayer coatings strongly depends on the bias voltage applied to the substrate. In ref. [42], it was found that increasing the bias value from −70 V to −140 V leads to an improvement in surface morphology. This is explained by the fact that at a higher bias the condensed particles have greater mobility and occupy more stable positions on the surface.

### 3.2. Surface Morphology and Elemental Composition

**Figure 2:**
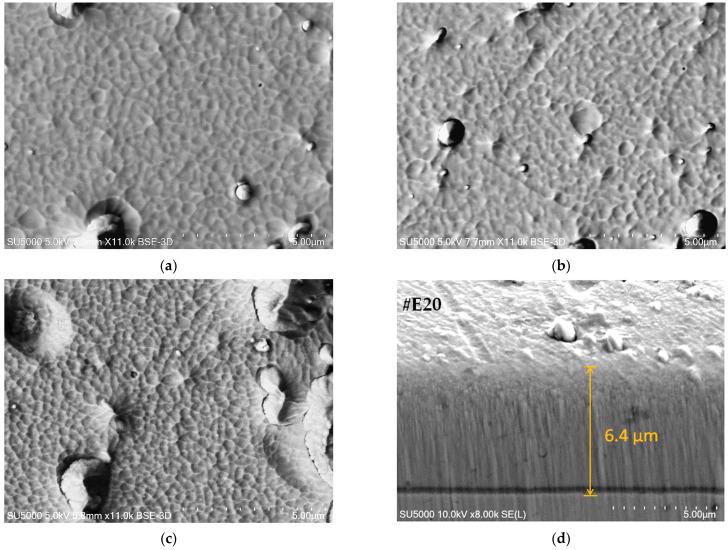
SEM images of the TiCN/ZrCN multilayer coatings: surface morphology of the samples (**a**) #E19, (**b**) #E20 and (**c**) #E21, respectively; (**d**) typical cross-sectional morphology.

Scanning electron microscopy (SEM) as well as atomic force microscopy images provides direct information on the state of the coating surface. Figure 2 shows SEM micrographs of the surface morphology of TiCN/ZrCN multilayer coatings at a high magnification (×11,000). The SEM examination confirmed that the coatings are dense, without the presence of cracks. The micrographs show the most characteristic defects for the cathode arc evaporation technology without filtration, such as droplets ejected from the molten material of the cathode, which solidify on the coating surface, and craters formed by mechanically detached droplets from the surface. During the formation of carbonitrides, the quantity and size of the defects change depending on the bias voltage [14] and the ratio of the reactive gases nitrogen and methane. Coatings with a lower carbon content have a coarser grain structure (Figure 2a,b), while the coating with the highest carbon content has a finer grain structure with the existence of larger surface defects (Figure 2c). The results are in good agreement with the AFM analysis performed, where the highest average roughness was calculated for this sample. Since the coatings were obtained under the same technological conditions, it can be assumed that an increase in the concentration of methane in the gas mixture and a proportional decrease in nitrogen during the deposition of the modulated structure lead to an increase in droplet size and a rougher surface.

In the Hitachi IM4000Plus ion etching system (Hitachi, Tokyo, Japan), a low-energy Ar+ ion beam was used to form the cross-section without applying mechanical stress to the sample. After the etching procedure, the specimen together with the holder was mounted in the SEM chamber for cross-sectional analysis. Scanning electron microscopy (SEM) images of the cross-section of the TiCN/ZrCN multilayer (Figure 2d) showed that the coatings had a dense columnar microstructure with grains extending from the interface to the surface. The total thickness of the coating measured in the cross-section by SEM is about 200 nm (deviation 3%) less than that determined by the Calotest method. A well-defined boundary between the substrate and the coating was observed for all samples. In general, examination of the cross-sections did not reveal any internal cracks through the coatings reaching the substrates. This suggests that the interfaces between the layers have a strong enough bond that prevents the formation and propagation of dislocations and cracks.

**Figure 3:**
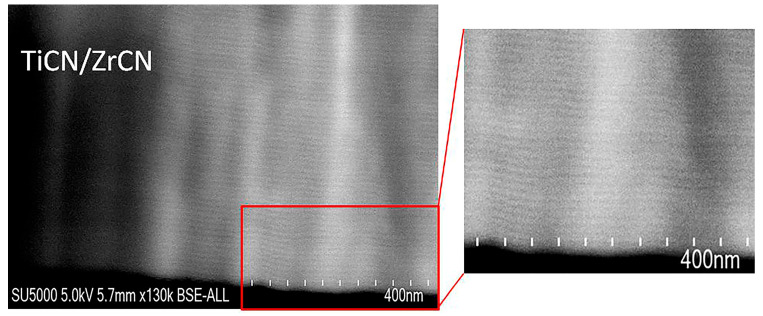
High resolution SEM image of cross-section the TiCN/ZrCN multilayer coating obtained at a magnification of 130,000×.

The high resolution cross-sectional micrograph of the TiCN/ZrCN multilayer coating is shown in Figure 3. As can be seen from the SEM micrograph, the multilayer structure has a uniform periodicity, but not very clearly visible interfaces between the individual nanolayers in the modulated structure. This is due to the poorer resolution of this technique compared to transmission electron microscopy in identifying very thin nanolayers along the cross-section of the sample [43]. 

This conclusion is supported by the results obtained in our previous work [37], where clearly distinguishable boundaries between individual nanolayers were observed, due to the larger thickness of the bilayers (Ʌ = 32 nm). Based on the calculated deposition rates of the individual monolayers of TiCN and ZrCN and the results obtained from the SEM analysis of the cross-section, it was found that the average value of the bilayer thickness is 12 nm. The thicknesses determined for the TiCN (dark stripe) and ZrCN (bright stripe) sub-layers were about 5.0 nm and 7.0 nm, respectively, which are in good agreement with the expected values. The difference in the sub-layer thickness found is due to a higher current applied to a zirconium target compared to a titanium target.

**Figure 4:**
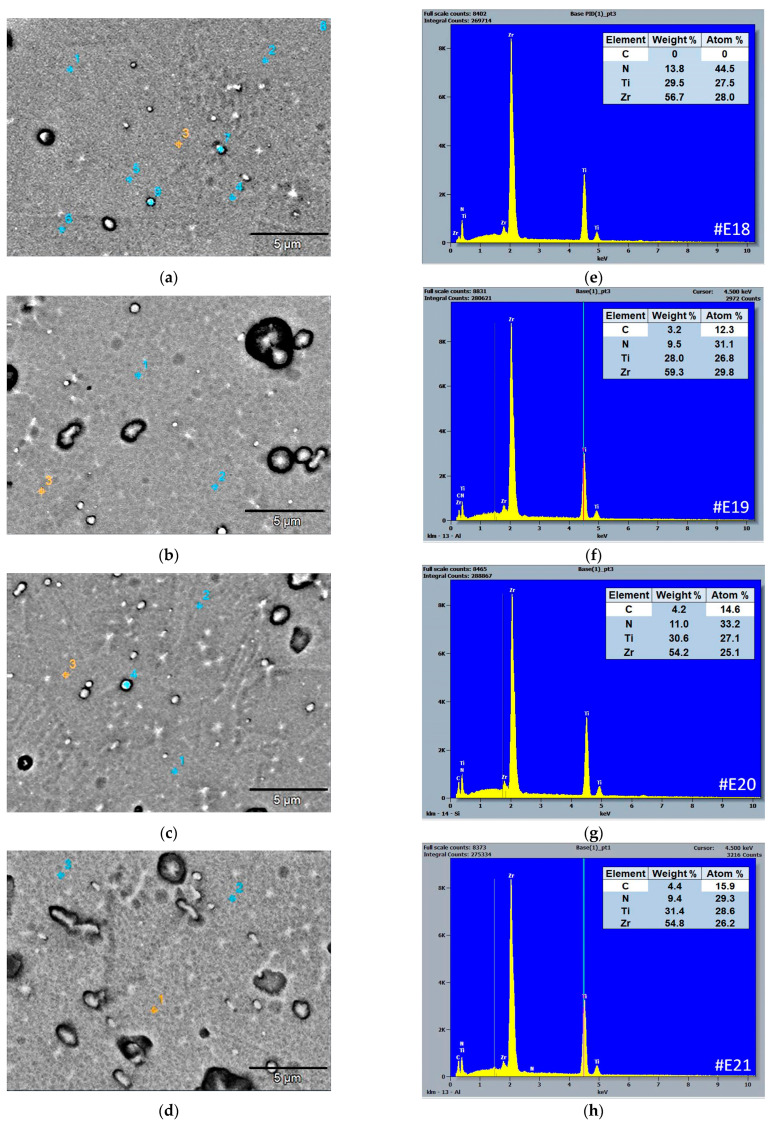
SEM surface morphology images of the TiCN/ZrCN multilayer coatings, obtained in BSE mode: (**a**) #E18, (**b**) #E19, (**c**) #E20, (**d**) #E21; corresponding EDS spectra with calculated elemental composition (**e**–**h**).

Figure 4a–d shows the SEM micrographs of the surface morphology, the multilayer TiCN/ZrCN coatings obtained in backscattering (BSE) mode, and the points where the EDS spectrum was recorded (encircled in red). The corresponding EDS spectra with calculated elemental composition are presented in Figure 4e–h. As can be seen from the results of the EDS analysis of the coatings #E18, #E19, #E20 and #E21, they all have a sub-stoichiometric composition. It should be noted that during scanning of the defects, variations in elemental composition (the presence of small amounts of oxygen) were detected in them. No significant difference was found in determining the atomic concentration of the elements when scanning the cross-section of the samples. A strict dependence of the carbon content on the flow rate of CH4 gas was established in the investigated multilayer coatings at a fixed total working pressure.

### 3.3. Structural Analysis

**Figure 5:**
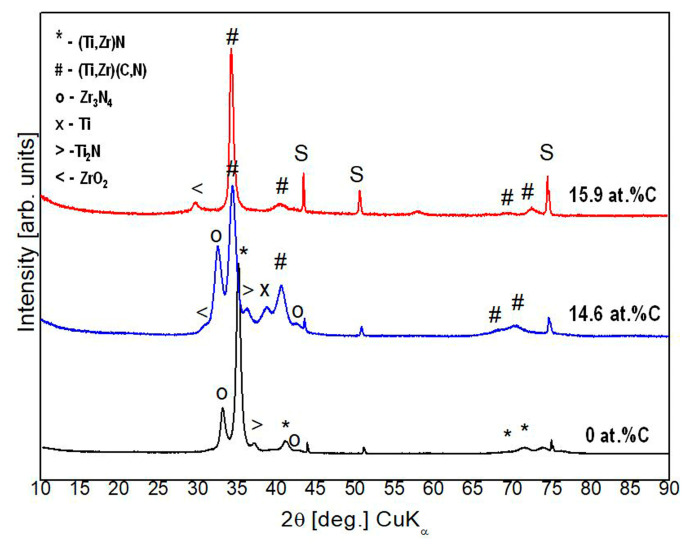
X-ray diffraction patterns of multilayer coatings with different carbon contents: black—0 at.%C (#E18), blue—14.6 at.%C (#E20), and red—15.9 at.%C (#E21); S—substrate.

Powder diffraction patterns of the samples are presented in Figure 5. In all cases, sharp peaks of the substrate at approx. 43, 52, and 75°2θ are observed. Analysis of the phase composition revealed that the coating layers consist of several phases. In the case of the TiN/ZrN multilayer, the diffraction pattern shows a high-intensity peak corresponding to the (111) plane of an fcc-phase. The unit cell parameter calculated for this phase is 4.4634(7)Å, and the mean crystallite size is 14 nm. The obtained parameter falls between the corresponding parameters of TiN and ZrN, indicating a mixed metal content. According to Vegard’s law, the composition of this phase is estimated as (Ti_0.6_ Zr_0.4_)N, close to that in TiZrN_2_ (PDF 01-077-3008). The other peaks of this phase show lower intensities than those of the referent powder pattern, indicating a strong preferred orientation of the film within the <111> direction. Formation of the two-phase state of TiN and ZrN with the development of the same orientation was observed in [44] for multilayer coatings of TiN/ZrN with different bilayer thicknesses. In another publication [45], the strictly preferred (111) orientation has also been detected for TiZrN coatings. A second phase of the TiN/ZrN coating (#E18) was identified as Zr_3_N_4_ (PDF 01-072-8064). The unit cell parameter of this phase is 6.701(2)Å and its mean crystallite size is 11 nm. The (111) peak of Ti_2_N (PDF 01-072-8064) is seen around 37 °2θ, and the determined parameters for this tetragonal phase are a = 4.138(6)Å, c = 8.82(1)Å. The mean crystallite size for Ti_2_N is again 11 nm. A similar phase composition is observed for the coating #E20 with 14.6 at.% carbon content. In this case, the peaks associated with the fcc-TiN type phase (PDF-01-083-8030) are shifted towards the lower angle compared to the corresponding peaks detected in TiN/ZrN. This shift is due to the solid solution effect caused by carbon incorporation in the (Ti,Zr)N crystal structure, suggesting the phase has a composition (Ti,Zr)(C,N). Similar slight shifts of the CrN (111) diffraction peaks towards a lower diffraction angle have been observed for CrCN/ZrCN nanomultilayer coatings with increases in the C_2_H_2_ flow ratio [35]. The unit cell parameter for this phase is 4.679(1) Å, and the mean crystallite size is estimated to be around 12 nm. It should be mentioned that the intensity ratio of (111)/(200) peaks for this case is lower than that in the pure TiN/ZrN, which advocates for some changes in the morphology of the coatings upon carbon incorporation. The presence of Zr_3_N_4,_ Ti_2_N, Ti, and traces of ZrO_2_ were also detected in this coating. The traces of ZrO_2_ are absent in the TiN/ZrN coating formed in a nitrogen atmosphere with 99.9999% purity, as can be seen in Figure 5. These traces appeared during the deposition of the TiCN/ZrCN coatings after the introduction of methane with 99.995% purity into the chamber, resulting in the presence of traces of oxygen. These minimal amounts are sufficient for the formation of the ZrO_2_ oxide phase, since Zr has a greater affinity for O_2_ than Ti. This is further evidenced by the fact that the ZrO_2_ peak becomes more prominent with increasing methane flow.

The diffraction pattern of the coating with the highest carbon content (15.9 at.%) shows a strong (111) peak of the fcc-(Ti,Zr)(C,N) phase, with unit cell parameter 4.680(1)Å, and mean crystallite size 10 nm. The intensity ratio of the (111)/(200) peaks being the highest among the studied coatings is an indication of the highly preferred orientation of this coating. The only additional phase in this coating is a trace-content ZrO_2_ phase, which may be due to some surface oxidation. The (111) preferred orientation is expected in thick coatings [46] deposited at relatively high substrate temperature due to the dominance of strain energy over surface energy.

**Figure 6:**
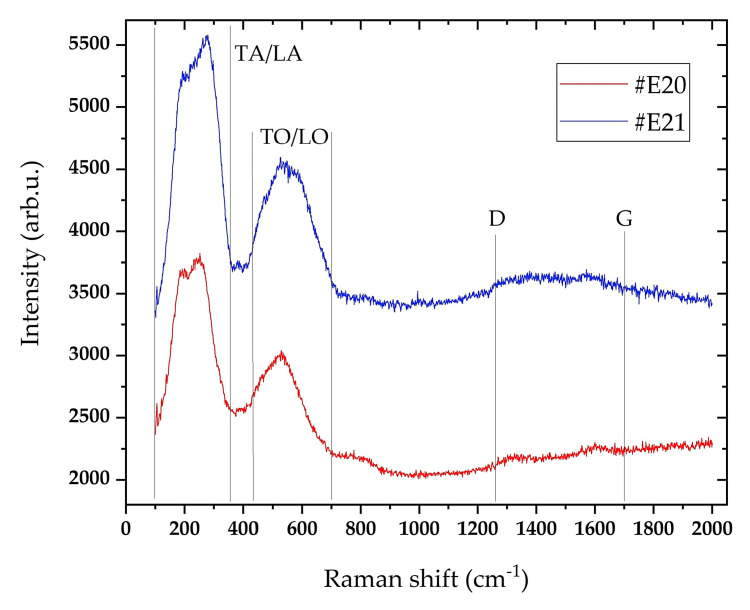
Raman spectra of the TiCN/ZrCN multilayer coatings: Red line—14.6 at.%C; Blue line—15.9 at.%C.

Information about the nature of the carbon bonds was obtained by Raman spectroscopy, which is one of the preferred methods for structural analysis of thin carbon-containing layers. Figure 6 presents Raman spectra of the TiCN/ZrCN multilayer coatings with 14.6 at.% (#E20) and 15.9 at.% (#E21) carbon content, for which two frequency ranges are detected. In the first frequency range from 150 to 700 cm^−1^, there are typical peaks associated with TiCN and ZrCN, arising from the transverse and longitudinal acoustic modes TA/LA and the transverse and longitudinal optical modes TO/LO [47,48]. In the studied coatings, no change in the position of the Raman peaks was observed, since they were deposited at the same substrate bias (−80 V). The peaks of both samples have similar widths, as the peaks of #E21 have a higher intensity. In the second spectral range, in the interval from 1250 to 1700 cm^−1^, weakly pronounced D and G bands with a maximum intensity at 1360 cm^−1^ and 1580 cm^−1^, respectively, were detected. The same broad bands corresponding to D and G peaks with a similar position were observed for another carbonitrides coating [22,28]. These peaks indicate the presence of disordered amorphous carbon (D) and graphitic carbon (G) in TiCN/ZrCN. The Raman spectrum locked between D and G bands has weak peaks, which are more pronounced in sample #E20. The calculated intensity ratio (I_D_/I_G_) of 0.96 and 0.99 for the samples #E20 and #E21 shows that the coatings have a composite structure, consisting of crystalline (Ti,Zr)(C,N) with the presence of amorphous carbon. In a number of publications, it was found that the position and intensity of the peaks in the second spectral range depend on a number of factors, such as high bias being applied to the substrate during the coating deposition [49] and most often, such peaks are observed in a combination of a high applied bias voltage with a high carbon content [50,51].

### 3.4. Mechanical Properties

#### 3.4.1. Nanoindentation Results

Samples with the coatings deposited on polished HSS substrates with a diameter of 20 mm, a thickness of 5 mm and an average surface roughness of Ra = 12 nm were used to investigate the mechanical and tribological properties. Nanoindentation was carried out at loads ranging from 20 mN to 500 mN to observe the change in hardness with depth and determine the loads at which the measurements are relevant to the properties of the coatings.

**Figure 7:**
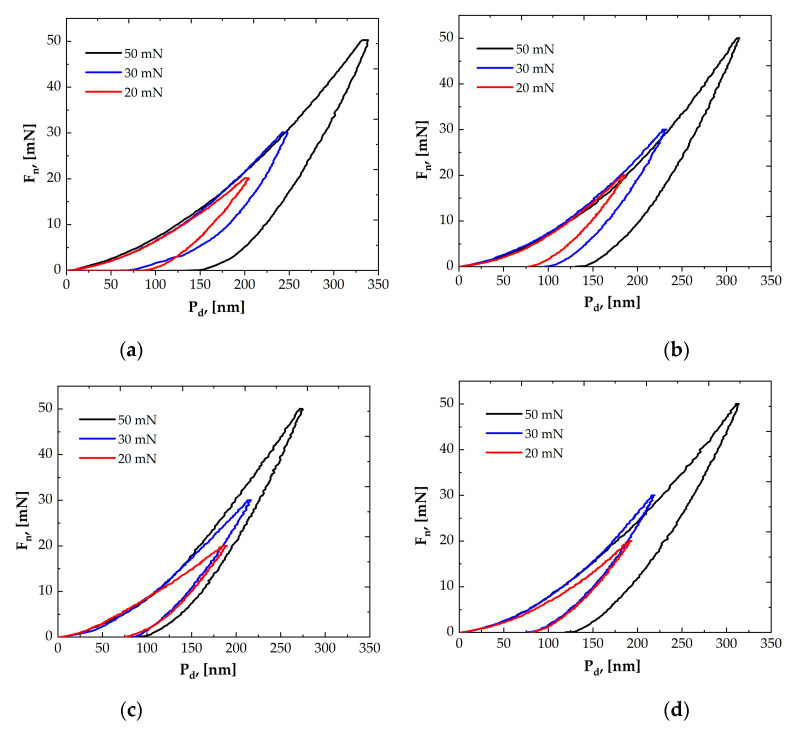
Load–displacement curves as a function of the indenter penetration depth for the coatings with different carbon contents: (**a**) 0 at.%, (**b**) 12.3 at.%, (**c**) 14.6 at.%, and (**d**) 15.9 at.%.

The measurements were performed in the “loading–unloading” mode. During the test, the indenter is dynamically loaded to the selected force, followed by a holding time of 5 s, and then released. When the diamond tip of the indenter enters a defect below the surface of the coating, the load–unload curve deviates from the ideal shape during the loading cycle (“pop-in” event), leading to a change/decrease in the hardness and elastic modulus values and the appearance of cracks. Most often, these events often occur in hard and brittle coatings [52,53]. The load–penetration depth curves of the multilayer TiN/ZrN and TiCN/ZrCN coatings are shown in Figure 7a–d. The forces of 20, 30, and 50 mN are presented in the graphs, as similar high hardness values were observed in this range where ten indentations were made for each load. Analysis of the curves showed that at the same loads, the smallest penetration depth was found in sample #E20 with a carbon content of 14.6 at.%, which is an indication of the best resistance to plastic deformation. No “pop-in” events were detected in the coatings within the nanoindentation loading range, suggesting that no bending-induced cracks occurred.

The nanoindentation results showed that all the tested TiCN/ZrCN coatings had improved hardness in the load range 20–500 mN, compared to TiN/ZrN. The high hardness values of 25–27 GPa, obtained under the maximum load of 500 mN are suggestive of the well-ordered structure of the coating. When calculating the hardness, only values at the maximum penetration depth of the indenter not exceeding 10% of the coating thickness were taken into account in order to avoid the influence of the substrate. The resulting values of nanohardness (H), modulus of elasticity (E), maximum indentation depth (h) and elastic recovery (We) are listed in Table 2.

Initially, with the addition of methane, an increase in hardness and modulus of elasticity was observed for #E19 compared to #E18. As can be seen from the Table 2, the highest average values of hardness of 40 GPa and elastic modulus of 450 GPa were obtained for the coating #E20 with the carbon content of 14.6 at.%. The improvement in hardness with an increase in the carbon content is due to the solid solution effect as was determined by the XRD analysis above. In individual cases, maximum hardness values of 49 GPa were measured for this sample. A decrease in hardness and modulus of elasticity to 36 GPa and 390 GPa, respectively, was observed for the multilayer coating #E21 with the highest carbon content (15.9 at.%), obtained at the nitrogen/methane flow rate of 330/140 sccm.

**Figure 8:**
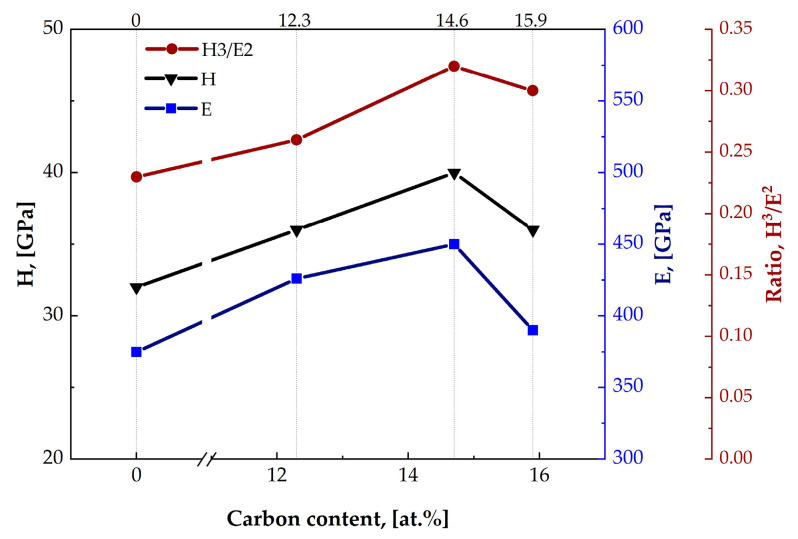
Dependence of the H, E and H^3^/E^2^ values on the carbon content in the coatings.

The best values of the plasticity index (H/E), representing the resistance to elastic stress to failure, resistance to plastic deformation (H^3^/E^2^) and elastic recovery (We) were determined for the samples #E20 and #E21. These results suggest better resistance of these coatings to destruction and wear. However, the sample #E21 combines a high hardness of 36 GPa with a lower modulus of elasticity (390 GPa), which implies greater resistance to cracking. The presence of multiple interfaces between nanolayers in multilayer coatings acts as a barrier to crack propagation, improving the toughness of the structure. The presence of amorphous carbon contributes to the flexible redistribution of mechanical stresses as compared to the crystalline phases, preventing brittle failure of the coatings. Similar behavior of mechanical parameters has been reported in other publications [35,54], where a reduction in hardness with increasing carbon content was established. Dependence of the average values of H, E and H^3^/E^2^ on the carbon content in the studied coatings is presented in Figure 8. Comparison of the obtained curves with those reported for TiAlCN/TiAlN/TiAl multilayer coatings [20] showed that they have similar trends. The difference was found in the carbon content (12.4 at.%) at which a change in the course of the curves occurs towards the lower values of the mechanical parameters. Analyzing the results obtained by different authors, it can be concluded that the maximum values of mechanical parameters in multilayer coatings are observed at different carbon concentrations depending on the developed deposition technology and the chosen coating architecture.

#### 3.4.2. Scratch Test Results

**Figure 9:**
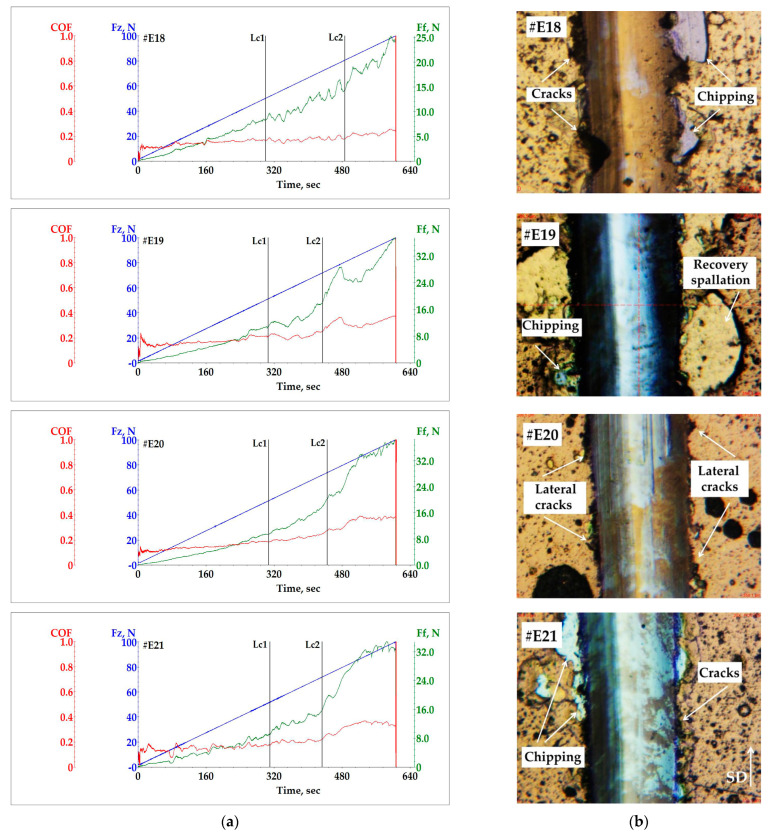
Scratch test results of the multilayer TiN/ZrN (#E18) and TiCN/ZrCN coatings (#E19–#E21): (**a**) evolution of friction force and coefficient of friction; (**b**) optical micrographs with determined Lc2 damages.

The scratch tests were carried out under s progressively increasing load from 1 N to 100 N and the following conditions: increasing rate 10 N/min, sliding distance 3.0 mm and total test time 600 s. The procedure applied is in accordance to International standard ASTM C1624-05 [55]. A Rockwell C diamond stylus with a radius of 200 μm, which is recommended for scratch tests at coating thicknesses up to 30 μm, was used. Three scratch tests were conducted across the coating surface for each sample to determine the critical failure loads (Lc) and their nature. Figure 9 presents the results of the adhesion strength tests of the TiCN/ZrCN multilayer coatings based on a complex analysis of the graphical data and microscopic examination of the resulting traces. For comparison, TiN/ZrN was also studied to evaluate the influence of carbon incorporation into the crystal structure on the adhesion properties of the coating. Figure 9a presents the evolution of the friction force (Ff) and the friction coefficient (CoF) with a progressive increase in the normal force (Fz) as a function of the scratch time. The friction force data are more reliable than the acoustic emission (AE) signals when analyzing damages in hard coatings after scratch testing. This is explained by the fact that when the indenter passes through surface defects, false AE signals can occur without causing damages to the coating. The scratch trace can be divided into three sections, as seen from the graphs. The experiments conducted showed that during the scratch test, the friction force is stable and increases smoothly as long as the adhesion is good. The first change in Ft is an indicator of the appearance of cohesive damage in the coating (Lc1). All the samples tested showed good cohesive properties, with the first failures being registered at relatively high values of Fz. The lowest value of Lc1 was obtained for the sample #E18 (48 N), and the highest for the sample #E21 (52 N). The evolution of the friction force and friction coefficient proceeded in a similar manner for all multilayer coatings until the first failure (Lc1) occurred, with the calculated average values for Ft and CoF being 10 N and 0.18, respectively.

A stronger change in the frictional force occurs when the adhesion deteriorates with a linear increase in load (second section on the graphs). Figure 9b shows optical micrographs of the trace sections in which the characteristic damages of the coatings were observed, and the critical load Lc2 was determined as a function of the applied normal force.

In total, various mechanisms of adhesive damages (cracking, chipping and spallation) have been identified for the tested coatings at high values of normal force (Fz ≥ 70 N). The best resistance to damage was shown by the coating TiN/ZrN (#E18), for which a Lc2 value of 80 N was determined. For this coating, stronger failures such as cracks and chipping were observed at the edges outside and inside of the scratch trace. However, these damages did not lead to the complete exposure of the substrate during the scratch test. The calculated average values for Ft and CoF in the second section are 16 N and 0.2, respectively. With a further increase in the Fz, the density of the failures increases without complete destruction of the coating. Lower Lc2 values of 70–74 N were determined for the multilayer TiCN/ZrCN coatings. The observation revealed that all the coatings have damages on both sides of the trace, displayed by cracks, chipping and spallation. The observed partial wear of the coating inside the traces is more pronounced in sample #E19 with the lowest carbon content (12.3 at.%). The adhesive damages outside the trace are mainly presented by relatively large areas of spallation and very small areas of chipping. For the sample #E20 with 14.6 at.% carbon content in the multilayer structure, mainly damage in the form of small lateral cracks was observed. The calculated average values for Ft and CoF in this section were 19 N and 0.28, respectively. The behavior of Ft and CoF of the sample #E21 (15.9 at.% carbon content) is similar to the sample #E20. Additionally, the Ft and CoF lines in the first and the second sections of the graph exhibit a small wavy oscillation. According to the results obtained by AFM and SEM, sample #E21 has the greatest average surface roughness. Presumably, this oscillation occurs as the indenter passes through larger defects accumulated on the coating surface. Among the TiCN/ZrCN coatings, this coating demonstrates the lowest average values of 16 N and 0.21 for Ft and CoF in section Lc2. The subsequent increase in Fz for all coatings investigated led to an extensive enlarge of the damaged areas in the third section, resulting in substrate exposure (Lc3). The obtained high values of the loading force for the occurrence of cohesive and adhesive failures correlate well with the high values obtained for the elastic recovery, as presented above (see Table 2). For comparison, lover values of critical load have been reported for TiCN and boron carbon-nitride (BCN) single-layer coatings [56], where the best results of 38.41 N (Lc1) and 49.32 N (Lc2) have been achieved for BCN. Significantly lower critical loads of 27.76 N and 19.85 N are found for TiSiCN/ZrN and TiSiCN/ZrCN multilayer coatings with a high carbon content, obtained using multi-arc ion plating technology [31].

The study of the mechanical properties of TiCN/ZrCN showed a well-ordered structure with high values of H, H/E and H^3^E^2^, as well as a high level of adhesion strength, depending on the carbon content. The higher values of mechanical properties obtained for the samples #E20 and #E21 correlate with better crack resistance and higher fracture toughness, which suggests better wear resistance. This conclusion is supported by the XRD spectra presented above, which demonstrate a high level of crystallinity with a predominant orientation (111) in the multilayers.

### 3.5. Friction and Wear Characterization

#### 3.5.1. Friction Coefficient

Ball-on-disk tests were conducted in accordance with the international standard ASTM G99 [57]. A total sliding time of 16,000 s (39,809 revolutions equal to 500 m) was recorded during the tribological tests. The test duration was chosen in advance, so that the coating was not completely removed, in order to determine the wear resistance of the modulated coating structure.

**Figure 10:**
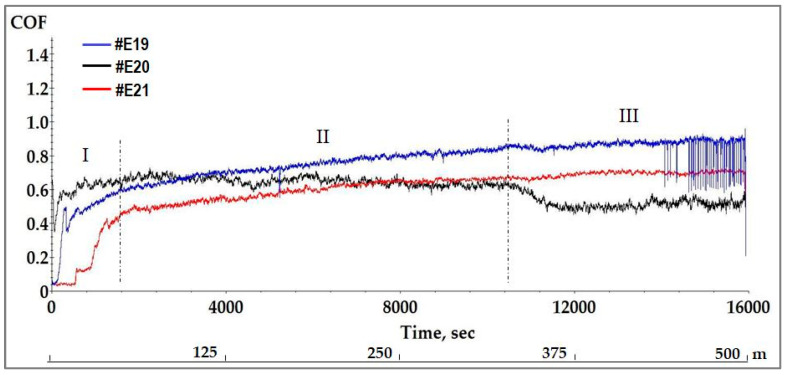
Evolution of the friction coefficient data recorded during the tribological tests.

After the testing, the evolution of the coefficient of friction (CoF) as a function of sliding time/distance (L) for all multilayer coatings was analyzed. The comparative graph with the obtained results is presented in Figure 10. On the additional line below the *X*-axis, the sliding distance is marked. The evolution of the coefficient of friction occurred in three stages: running-in (I), steady-state (II) and steady-state (III). The running-in stage lasts approximately 1600 s (50 m), after which a steady-state is maintained for samples #E19 and #E21 until the end of the test. The steady-state (II) for sample #E19 occurs earlier than for #E21 due to a lower surface roughness. The CoF in the running-in stage increases steeply from the initial value to about 0.56 (#E19) and 0.45 (#E21) at the end of first stage. The increase in the COF values is due to the initial contact interaction and contact stresses between the coating and the Al_2_O_3_ counterbody. A stable wear mechanism occurs (steady-state stage II) when a close contact between the counterbody and the coating is established. Subsequently, the CoF curves for both samples run parallel, with #E19 retaining higher coefficient of friction values at the end of the test. In the third section, the coefficient of friction of both coatings remains unchanged until the end of the test. The calculated average CoF values for samples #E19 and #E21 after the tribo-tests are 0.75 and 0.58, respectively. Sample #E20 showed a different behavior during the test and was characterized by a sharp change in the friction coefficient at stage III. A slightly wavy course of the friction coefficient curve is observed throughout the test period for this sample with a tendency to decrease the friction coefficient. Such a course may be due to unstable friction during a long fit between the coating and the counterbody. At the end of stage I, this coating showed the highest CoF value (0.64) and then throughout stage II, a slow decrease in the CoF to 0.56 was observed until reaching 25,950 revolutions (326 m). At the beginning of the third stage, a drop in the CoF to 0.45 was observed and then this value was kept until the end of the test. During prolonged friction of Al_2_O_3_ with a hardness of 19 GPa against a significantly harder TiCN/ZrCN coating (40 GPa), the surface of the counterbody is subjected to intense cyclic loading (39,809 revolutions), which leads to a characteristic wear mechanism. As a result of the interaction with the harder coating, the local stresses at the contact points exceed the fatigue limit of Al_2_O_3_. Microcracks formed under the surface grow and lead to the appearance of pitting in the material. This leads to a decrease in the real contact area in the tribo-pair and may be the reason for a change in the friction coefficient for sample #E20 in the third section of the curve. The assumption is also supported by the shape of the wear profile of the coating and the counterbody, presented below. Since the multilayer structures are deposited under the same technological conditions, behavior of the friction coefficient is mainly determined by the chemical composition of the coatings deposited at different ratios of the reactive gases and the formation of a transfer layer. All the TiCN/ZrCN coatings studied have a high critical adhesion force, as established above, and the higher friction coefficient obtained for sample #E19 may be due to the higher concentration of chipped particles during the tribo-test and the lower carbon content.

#### 3.5.2. Wear Resistance

**Figure 11:**
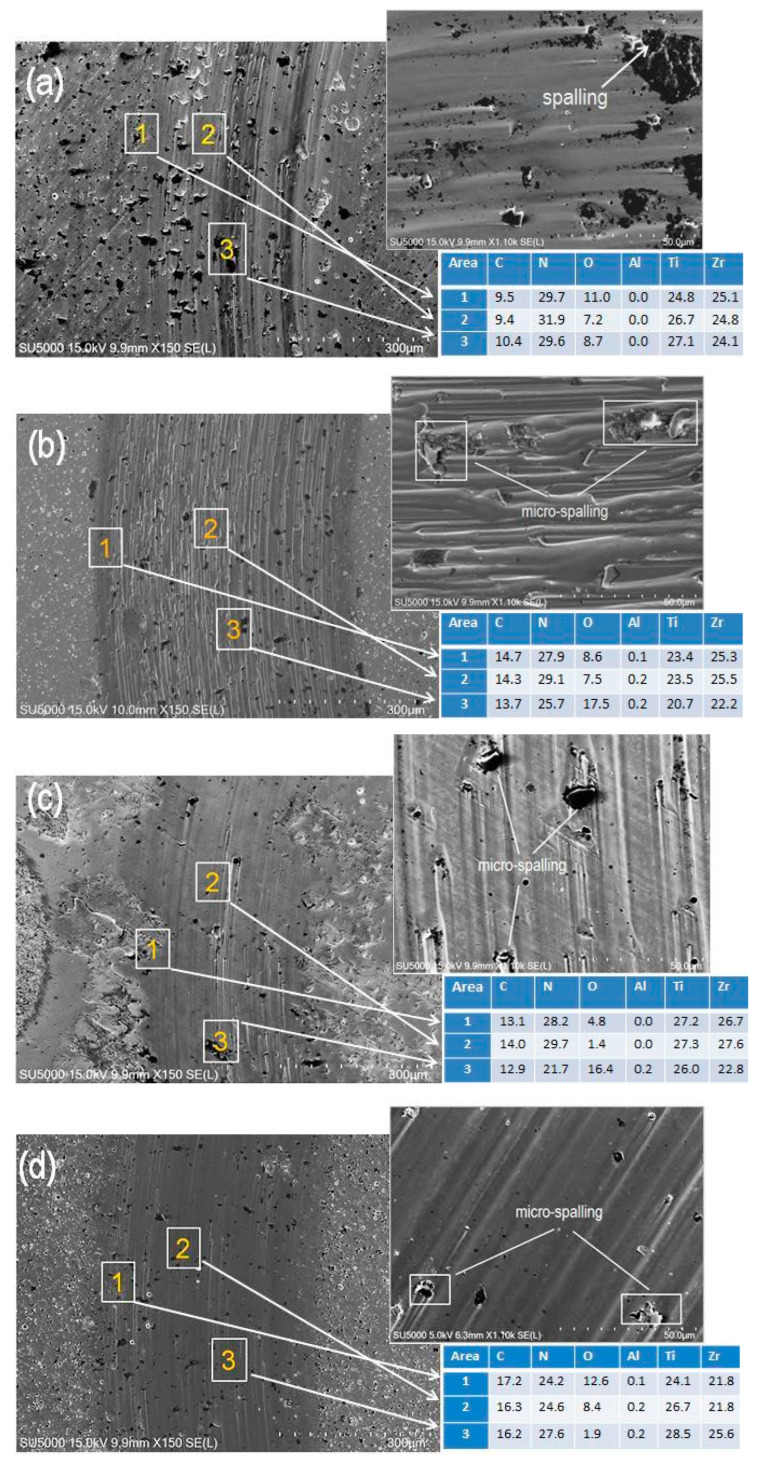
SEM micrographs of the wear tracks together with the higher magnification images (SE mode) and the elemental composition of the coatings in marked areas: (**a**) #E18, (**b**) #E19, (**c**) #E20 and (**d**) #E21.

The wear traces of all coatings were analyzed using SEM/EDS in order to determine the wear mechanisms and composition of the coatings after the tribo tests. The results of the SEM analysis are presented in Figure 11. On the wear track of the sample #E18 (Figure 11a), the presence of grooves and many areas of destruction in the form of spalled and adhered particles can be observed, which are clearly visible in the micrograph at higher magnification (×11,000). This indicates that an adhesion wear mechanism predominates for this coating. Similar tribological behavior was observed for a TiAlCN/TiAlN/TiAl (C = 0 at.%) multilayer coating in the work [20], where a Si_3_N_4_ counterbody was used for the tests. SEM examination of the track on the sample #E19 with 12.3 at.% carbon content (Figure 11b) revealed a significantly lower amount of spalling areas and a change in the wear mechanism. In this case, a dominance of the abrasive wear mechanism was observed. Abrasive wear in the tracks was observed for the coatings #E20 and #E21. On the track micrographs of the coatings #E20 and #E21 (Figure 11c,d) there are clearly visible grooves and a very small number of micro spallings. Due to the large number of defects on the surface of #E20, the outer boundaries of the track are non-uniform in some sections. This sample showed larger variations in track width. After the tribo tests, EDS analysis was performed in different areas on the tracks to determine the chemical composition. The results obtained in the marked areas showed that the composition of the main elements in the coatings #E20 and #E21 is in good agreement with the composition determined before the tribological tests (see Figure 4). A higher presence of oxygen and carbon was found on the tribo-track in the chipped areas of samples #E18 and #E19, due to the transfer of the worn material from the Al_2_O_3_ counterbody and the adsorption of carbon dioxide from the environment.

After testing the wear tracks, it can be assumed that the multilayer coatings #E18 and #E19 have the most pronounced wear. Since all TiCN/ZrCN coatings have a high critical adhesion force, the higher friction coefficient of #E19 is due to the lower carbon content in this coating and the mixed wear mechanism.

The investigation of the tribo-track width and the determination of the wear depth of the coatings were carried out in two ways: using the Compact Platform CPX (MHT/NHT) micro-scratching equipment in scanning mode [58] and using the Calotest CAT^2^ calo-wear equipment.

**Figure 12:**
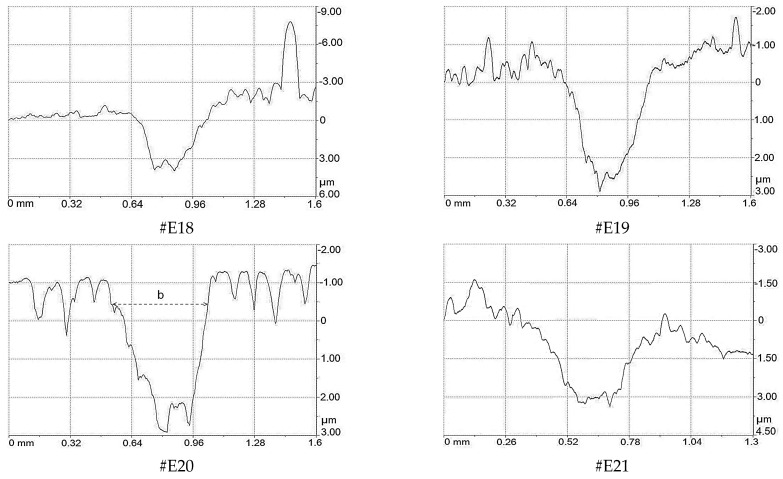
Surface profiles of the wear track on the TiN/ZrN (#E18) and TiCN/ZrCN (#E19, #E20, #E21) multilayer coatings; b—width of the wear track.

Figure 12 presents the profiles of the tracks obtained at a constant load of 0.03 N and an indenter scanning speed of 0.1 mm/min across the wear track at a distance of 1.6 mm. The width of the tracks was determined at 20× magnification and the maximum wear depth for all tested coatings was calculated using Anton Paar software version 6.0.35.

**Figure 13:**
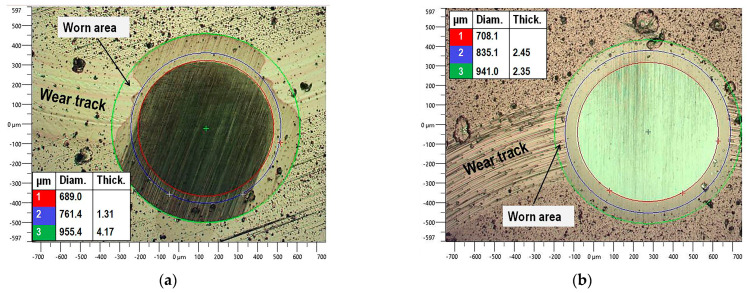
Optical images of calo-wear test results for: (**a**) TiN/ZrN and (**b**) TiCN/ZrCN (#E21) coatings.

The wear depth of the coatings obtained from the profiles was compared with the wear depth calculated after the calo-wear tests. In this case, a spherical crater (calotte) was formed in the middle of the track. The depth of wear was determined using the same Calotest software Version 9.0.12 as used for the thickness calculation [38,59]. The value of the coating wear depth in both cases was determined using averaged data obtained in four evenly distributed locations of the tribological track [60]. Figure 13 presents optical images of craters formed on the tribo-tracks for the coating #E18 with a carbon-free composition (Figure 13a) and for the coating #E21 with the highest carbon content (Figure 13b). The worn area is locked between the green and blue lines and the calculated wear depth is 4.17 μm and 2.35 μm for TiN/ZrN and TiCN/ZrCN, respectively. The results obtained by both methods for all coatings are in good agreement, with a deviation not exceeding ±100 nm.

The surface of the wear scar on the balls was observed using an optical microscope at 10× magnification, as the formation of a transfer layer on the ball surface significantly affects the wear mechanism and therefore the wear behavior of the coatings.

**Figure 14:**
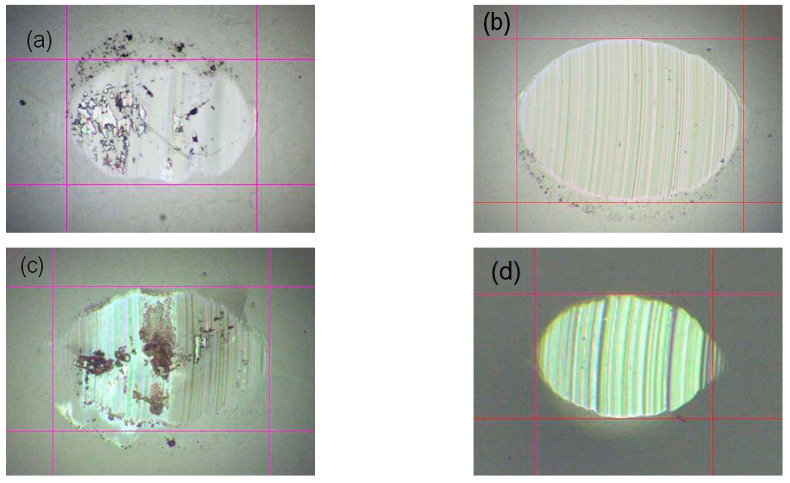
Optical images of the Al_2_O_3_ counterbody surface after interaction with the coatings: (**a**) #E18, (**b**) #E19, (**c**) #E20 and (**d**) #E21.

Figure 14 shows the optical images of the wear scar on Al_2_O_3_ ball after the wear test with the indicated dimensions. The dimensions obtained of the resulting wear scars are smaller on the surface of the Al_2_O_3_ sphere (Figure 14a) after testing the E18 sample, since due to the smaller thickness of this coating, the duration of the tribological test was reduced to 20,000 revolutions. On the worn surface of the ball there is the presence of a transfer layer, which has been observed by other authors [61,62] for nitride coatings. Inspection of Al_2_O_3_ surfaces after testing the multilayer carbonitrides up to 39,809 revolutions showed that the dimensions of the abraded surfaces decrease with increasing carbon content in the coatings. The wear scars are clean after testing the samples #E19 (Figure 14b) and #E21 (Figure 14d), without the presence of particles transferred from the coating.

A transfer layer was observed on the left side of the abraded surface of Al_2_O_3_ (Figure 14c) after testing of the sample #E20. A closer examination showed that the abraded surface of the Al_2_O_3_ ball had concave areas containing material transferred from the coating. We assume that these were formed due to fatigue of the ball material during prolonged pressure of the counterbody on the coating with much higher hardness and elasticity (see Table 2).

The wear rates (*W_R_*) of the coatings and the balls were calculated from the worn volumes using Archard’s wear equation [63]:

(1) $$W_{R}=\frac{V}{F_{z}.\;\;L}\;,$$

where *V* is the worn volume (mm^3^), *F_Z_* is the normal load (N) applied on the ball and *L* is the total sliding distance (m).

To determine the wear rate of the coatings, the volume (*Vc*) losses was calculated using the formula [35,64]:

(2) $$V_{C}=\frac{t}{6b}\left(3t^{2}+4b^{2}\right)2\pi r,$$

where *t* is the depth of wear track (mm), *b* is the width of the wear track (mm) and *r* is the radius of the wear track (mm).

The wear scar profiles of the counterbody were measured in two orthogonal directions and then the results were averaged. The following equation [15,65] was used to calculate the volume losses of the counterbody (*V_b_*):

(3) $$V_{b}=\frac{\pi \;d^{4}}{64R}=\;\frac{\pi \;A^{3}B}{64R},$$

where *d =* wear scar diameter (mm), and *R* = original ball radius (mm). Since the wear scar of the ball after the test has an elliptical shape and not a spherical one, then *d*^4^ = *A*^3^*B*, where *A* is the smaller diameter and *B* is the larger diameter.

A smaller worn volume was determined for samples #E20 and #E21, suggesting better resistance of these coatings to fracture and wear due to the stronger resistance to plastic deformation, as presented in Table 2 above. The resulting average values of wear characteristics for the different friction pair are summarized in Table 3.

As shown in the table above, an increase in carbon content leads to a reduction in the wear rate of the coatings and the counterbody. The analysis of the obtained results showed that the multilayer TiN/ZrN coating (#E18) demonstrated the highest wear rate. However, this wear rate is lower than those reported in other publications [44,66] for the TiN/ZrN multilayer coatings tested under identical conditions at room temperature. The multilayer TiCN/ZrCN coating (#E21) with the highest carbon content of 15.9 at.% exhibited the lowest wear rate of 3.16 × 10^−6^ mm^3^/N·m. The result obtained is better than that reported for TiSiCN/ZrCN [31], but slightly lower than those for CrCN/ZrCN [35]. It should be noted that the tribological results obtained are valid for the specific friction counterbody used under the selected testing condition.

## 4. Conclusions

TiCN/ZrCN nanomultilayer coatings with a modulation period of 12 nm were successfully obtained using a conventional cathodic arc evaporation system. Compared to TiN/ZrN, the multilayer TiCN/ZrCN coatings showed improved tribo-mechanical properties (higher adhesion, hardness and fracture toughness), as follows:

All tested samples showed similar adhesion/cohesion properties, with the first and second failure values (Lc1, Lc2) falling within 48–52 N and 70–74 N, respectively, indicating that the N_2_/CH_4_ flow ratios used did not result in significant changes in adhesion strength. Increasing the methane flow rate initially led to an increase in the hardness and modulus of elasticity, but at a maximum flow rate of 140 sccm, these values decreased. The highest average hardness of 40 GPa was obtained for the coating with 14.6 at.% carbon content, deposited at a N_2_/CH_4_ flow rate of 370/100 sccm. The average coefficient of friction was lower for the coatings with 14.6 at.% and 15.9 at.% carbon content, measuring 0.56 and 0.58, respectively. The lowest wear rate of 3.16 × 10^−6^ mm^3^/N·m under dry sliding conditions was exhibited by the multilayer coating with 15.9 at.% carbon content, deposited at a N_2_/CH_4_ flow rate of 330/140 sccm.

The results obtained suggest that the developed coatings are well-suited for industrial applications. Nanomultilayer TiCN/ZrCN coatings, characterized by a high number of alternating bilayers with a short modulation period and a balanced combination of tribo-mechanical properties are particularly effective for high-speed machining of non-ferrous metals, as they prevent material accumulation on cutting tools. Future research will focus on optimizing the balance between the mechanical and tribological properties of the TiCN/ZrCN nanomultilayer, building upon the findings of the present study.

## Figures and Tables

**Table 1:** Deposition conditions of TiCN/ZrCN multilayer coatings.

Position/ Sample	Process Phase	N_2_ Flow Rate, Sccm	CH_4_ Flow Rate, Sccm	Ti Target Current, A	Zr Target Current, A	Deposition Time, min
1	Ti	460	0	70	0	3
2	TiN	460→470	0	70→110	0	5
3	TiZrN	470	0	110	120	5
#E18	TiN/ZrN_ml	470	0	110	120	53
#E19	TiCN/ZrCN_ml	390	80	110	120	53
#E20	TiCN/ZrCN_ml	370	100	110	120	53
#E21	TiCN/ZrCN_ml	330	140	110	120	53

**Table 2:** Mechanical properties of the coatings based on nanoindentation tests.

Sample [#]	Coating	d [μm]	H [GPa]	E [GPa]	h [nm]	H/E [-]	H^3^/E^2^ [GPa]	W_e_ [%]
HSS	Substrate	5 × 10^3^	12	260	1000	0.046	0.026	-
#E18	TiN/ZrN_ml	5.4	32	375	204	0.084	0.23	55.8
#E19	TiCN/ZrCN_ml	6.2	36	426	318	0.085	0.26	58.7
#E20	TiCN/ZrCN_ml	6.4	40	450	228	0.089	0.32	64.5
#E21	TiCN/ZrCN_ml	4.8	36	390	315	0.092	0.31	60.4

**Table 3:** Tribological properties of tested coatings.

Sample	Wear Track Width, *b* [mm]	Wear Track Depth, *t* [mm]	Worn Volume, *V_c_* [mm^3^]	Wear Rate, *W_c_* [mm^3^/N·m]	Worn Volume, *V_b_* [mm^3^]	Wear Rate, *W_b_* [mm^3^/N·m]
#E18	0.386	0.0040	13.1 × 10^−3^	10.05 × 10^−6^	3.03 × 10^−4^	2.42 × 10^−7^
#E19	0.480	0.0032	12.7 × 10^−3^	5.09 × 10^−6^	8.64 × 10^−4^	3.46 × 10^−7^
#E20	0.430	0.0032	11.5 × 10^−3^	4.61 × 10^−6^	6.52 × 10^−4^	2.61 × 10^−7^
#E21	0.410	0.0023	7.90 × 10^−3^	3.16 × 10^−6^	5.59 × 10^−4^	2.24 × 10^−7^

## Data Availability

The original contributions presented in this study are included in the article. Further inquiries can be directed to the corresponding author.
